# Incidence, pathogens and antimicrobial resistance of blood and cerebrospinal fluid isolates from a tertiary neonatal unit in South Africa: A 10 year retrospective review

**DOI:** 10.1371/journal.pone.0297371

**Published:** 2024-01-19

**Authors:** Reenu Thomas, Claude Ondongo-Ezhet, Nini Motsoaledi, Mike Sharland, Michelle Clements, Sithembiso Velaphi

**Affiliations:** 1 Faculty of Health Sciences, Department of Paediatrics, Chris Hani Baragwanath Academic Hospital and School of Clinical Medicine, University of the Witwatersrand, Johannesburg, South Africa; 2 St. Georges, University of London, London, United Kingdom; 3 MRC Clinical Trials Unit at UCL, London, United Kingdom; Hawassa University College of Medicine and Health Sciences, ETHIOPIA

## Abstract

**Objective:**

To determine trends in incidence, etiology and antimicrobial susceptibility of blood and cerebrospinal fluid (CSF) culture confirmed infections in hospitalized infants in a large tertiary neonatal unit in South Africa.

**Methods:**

Single-center, retrospective review of laboratory records of bacteria and fungi, and their susceptibility profiles, isolated from blood and CSF of infants hospitalized in the neonatal unit at Chris Hani Baragwanath Academic Hospital, Johannesburg, South Africa, from 1^st^ January 2010 to 31^st^ December 2019. Laboratory data on isolates and their antimicrobial susceptibilities were collected. Coagulase-negative *Staphylococcus*, *Corynebacteria* and *Bacillus spp*. were excluded. Patient-level clinical and laboratory data were not available.

**Results:**

There were 8,319 significant isolates, giving an infection rate of 14.3/1000 patient-days. Infection rates increased from 12.0 to 15.7/1000 patient-days (estimated average yearly change 0.6[95%CI, 0.5–0.7];p = <0.001). Gram-negative infection rates increased from 4.3 to 10.8/1000 patient-days (estimated average yearly change 0.7[95%CI,0.6–0.8];p = <0.001). The 2 most commonly isolated Gram-negative organisms were *Acinetobacter baumannii* (44%) and *Klebsiella pneumoniae* (39%). Carbapenem resistance was seen in 31% of all Gram-negatives and increased over time (estimated average yearly change 4.8%[95%CI,4.2%-5.3%];p<0.001). Gram-positive infection rates decreased (estimated average yearly change -0.1[95%CI,-0.2– -0.05];p = <0.001). *Staphylococcus aureus* was the most common Gram-positive isolated. Rates of methicillin-resistant *Staphylococcus aureus* decreased from 91% to 55%(estimated average yearly change -2.8%[95%CI,-3.5%–2%],p< 0.001). Rates of fungal isolates decreased (estimated average yearly change -0.06[95%CI,-0.1 –-0.02]);p = 0.007). *Candida parapsilosis* (52%) and *Candida albicans* (35%) were the most common fungi isolated.

**Conclusions:**

There has been a marked overall increase in rates of blood and/or CSF infections, with an absolute increase in Gram-negative infections observed, replacing Gram-positive and fungal pathogens. Extended spectrum beta-lactamase Gram-negative isolates are being replaced by carbapenem resistance, with around one third of all significant Gram-negative isolates now carbapenem resistant. Research into hospital based novel treatment and prevention interventions for neonatal sepsis should be urgently prioritized.

## Introduction

Neonatal infections are a major cause of morbidity and mortality. A recent meta-analysis reported a global neonatal sepsis incidence of 28.2 cases/1000 live-births [1,2]. In low-to-middle income countries (LMIC) neonatal infections was reported to be highest over the last decade at 39.3 cases/1000 live-births. The incidence of healthcare-associated infections (HAI) varied between 15.2 and 62.0/1000 patient-days in neonatal units, with the highest burden in LMIC [1].

Globally, the most common pathogens responsible for neonatal infections are *Staphylococcus aureus* and *Klebsiella* spp. [1]. Group B *Streptococcus*, the leading pathogen responsible for early-onset neonatal sepsis (EONS) in high income countries (HIC), together with *Escherichia coli* (*E*. *coli*), account for approximately 70% of all EONS cases [2]. Organisms responsible for HAI include Gram-negative pathogens such as *Klebsiella spp*., *E*. *coli*, *Pseudomonas spp*. *and Acinetobacter spp*.; Gram-positive pathogens such as Coagulase-negative *Staphylococci* (CoNS) and *Staphylococcus aureus;* and fungi such as *Candida albicans*, *Candida parapsilosis* and *Candida auris* [3–7]. There are significant geographical variations in causative pathogen types, with Gram-negative organisms being more predominant in LMIC compared to HIC, where Gram-positive organisms predominate [6–9]. Additionally, in LMIC there is an overlap between pathogens typically responsible for EONS and those commonly identified in HAI, such as *Klebsiella spp*., *Staphylococcus aureus* and *Acinetobacter spp*., unlike in HIC where there tends to be a clear distinction in pathogen types causing EONS and HAI [10–13]. Data from HIC have shown a reduction in the incidence of HAI over time, with no significant differences in overall pathogen distribution [14].

A large proportion of organisms isolated in neonatal HAI are multidrug resistant organisms (MDRO) [14–18]. Most studies reporting on the prevalence of MDRO are from HIC [19–23], with few studies from LMIC [4,24–28]. A prevalence study conducted in three tertiary centers in India reported on high rates of antimicrobial resistance (AMR) among Gram-negative and Gram-positive organisms [4]. Another study reporting on the AMR profiles of Gram-negative bacteria from seven LMIC found that 54% of isolated bacteria demonstrated widespread carriage of resistance genes [16]. An observational study in neonates with clinical sepsis conducted across eleven countries, mostly LMIC, also reported high rates of AMR among Gram-negative pathogens [29].

Neonatal infections are a leading cause of mortality. Globally, the reported mortality from neonatal sepsis is 17.6%, mostly affecting preterm and very low birth weight infants [1]. Infants with culture positive sepsis have a higher mortality than those culture negative sepsis, with Gram-negative infections accounting for up to 76% of culture positive deaths [29]. Mortality rates are also reported to be higher in infants with MDRO [30,31].

The reasons for the HIC/LMIC differences in neonatal sepsis epidemiology are unclear as data on longitudinal trends in neonatal sepsis from LMIC are limited. The only available studies are single center and some multicenter cohorts across various continents, over a period of 1–2 years [4,16,32]. In this study we aimed to determine trends in incidence, etiology and AMR patterns of blood and cerebrospinal fluid (CSF) culture confirmed infections over a decade, in a single large tertiary neonatal unit from an upper middle-income country in Africa, serving many low-income groups.

## Materials and methods

### Study design and study population

This was a retrospective review of laboratory records of bacteria and fungi isolated from blood and CSF, and their susceptibility profiles, from infants admitted to the neonatal unit at Chris Hani Baragwanath Academic Hospital (CHBAH) from 1^st^ January 2010 to 31^st^ December 2019.

### Study setting

CHBAH is a public, tertiary hospital serving a population of approximately 2 million people in Soweto, a suburb in Johannesburg, South Africa. The hospital conducts approximately 20,000 of the 30,000 annual births in Soweto and is a medical and surgical referral hospital for government clinics in Soweto and surrounding district and regional hospitals. The 185 bedded neonatal unit at CHBAH consists of 18 intensive-care beds (level 3 nursery), 48 high-care beds (level 2 nursery), 100 standard-care and 19 kangaroo mother-care beds (level 1 nursery). The number of neonatal unit beds remained unchanged during the study period. The neonatal unit caters for infants born at CHBAH and surrounding maternity-obstetric units in Soweto, requiring admission, as well as infants born at surrounding district and regional hospitals, who have never left the facility, requiring specialized, tertiary care. The referral patterns remained unchanged during the study period. There are approximately 4000 admissions into the neonatal unit annually, of which around 5% are out-born. Indications for admission include both medical and surgical diagnoses. Surgical diagnoses comprise around 10% of all admission diagnoses. Approximately a third of admitted infants are Human Immunodeficiency Virus (HIV) exposed. Prematurity accounts for a major bulk of admissions, with a low birth-weight rate of approximately 20%. The overall mortality rate of infants admitted to the neonatal unit is approximately 12%, with prematurity being a leading cause of mortality, followed closely by sepsis. Overall mortality rates remained unchanged during the study period.

The unit protocol for the diagnosis and management of neonatal sepsis remained unchanged over the study period and were based on World Health Organization (WHO) criteria [33]. All infants with clinical symptoms/signs suggestive of infection at birth, on admission to the neonatal unit, or during hospital stay have blood samples taken for microbiologic culture. When an infant is suspected to have sepsis, one peripheral blood sample is collected for microscopy and culture, as well as other biomarkers, such as complete blood count and C-reactive protein (CRP). Cerebrospinal fluid (CSF) is collected for culture either at the time of the sepsis work-up or when there is a confirmed blood stream infection (BSI), provided the infant is clinically stable. Samples are collected from the unit by the laboratory messenger, who does hourly rounds, and taken to the laboratory for processing. In adherence to antimicrobial stewardship (AMS) principles, empiric antimicrobial therapy is started after collection of the culture specimen/s, except in cases where admission cultures are taken on patients referred from other facilities who have already been started on empirical antimicrobial therapy.

Infants with signs suggestive of infection at birth are started empirically on ampicillin and gentamicin. For suspected HAI, infants are started empirically on a combination of piperacillin-tazobactam and amikacin, or meropenem if deemed to be severely ill by the attending physician. This approach did not change during the study period. However, in recent years, with increasing cases of carbapenem resistant organisms seen in the neonatal unit, colistin may be added empirically in infants already on meropenem for 24–48 hours and showing clinical deterioration with signs of haemodynamic instability, severe metabolic acidosis or increased ventilatory support requirement.

The microbiology laboratory uses an automated continuous monitoring blood culture system for blood culture samples (BacT/Alert system, BioMerieux, Marcy l’Etoile, France). If bacterial growth is detected, a Gram stain is performed and the sample sub-cultured onto appropriate media and incubated overnight. For CSF samples, cell counts and gram staining are performed, and thereafter sub-cultured onto appropriate plates and broth. If the broth is turbid, a Gram stain is performed and the broth sub-cultured on the appropriate plate. Direct identification of Gram-negative bacteria is performed using the biochemical panel, API (Analytical Profile Index) 20E (BioMerieux, inc). The API 20NE (BioMerieux, inc) is used for identification of Gram-negative non-fermenters. For Gram-positive bacteria, biochemical tests such as the Staphyaurex^®^ (Murex Diagnostics Ltd., Kent, England) and Streptex™ latex agglutination tests (Remel Europe Ltd., Kent, England) are performed for direct identification. Further identification and antimicrobial susceptibility testing (AST), with minimum inhibitory concentrations (MIC), for all blood and CSF bacterial isolates are performed using either the Kirby-Baeur disc method or the automated system, Microscan, Siemens, USA. For yeast identification, the biochemical Auxacolor™ (BioRad Laboratories, Marnes-la-C0quette, France) system is used, and the E-test method is used for MIC testing. During the study period, the automated Vitek 2 (BioMerieux, inc) system was only available for yeast identification, as it was not big enough to accommodate bacterial samples. All identification and AST were interpreted according to the Clinical Laboratory Standards Institute standards, for the relevant year of study [34]. All changes in CLSI guidelines since 2010 were updated annually and applied accordingly at the laboratory, during the study period.

Microbiology results are communicated telephonically between the microbiologists and the neonatologists on a daily basis. If the culture is positive, antibiotics are tailored appropriately, once identification and susceptibility results are available. All carbapenem resistant infections are treated with colistin in combination with meropenem.

The neonatal unit at CHBAH has an infection prevention and control (IPC) program, with 2 IPC trained nurses. IPC policies include strict hand hygiene, transmission-based precautions, environmental cleaning, and AMS. Weekly AMS rounds with clinicians, microbiologists, infectious diseases specialists and the IPC nurses are conducted. Re-enforcement of IPC measures are implemented through ongoing training on hand hygiene practices, standard and transmission-based precautions, environmental hygiene, safe use of medical equipment, medication safety and antimicrobial stewardship practices. Both IPC and AMS practices remained unchanged during the study period.

### Data collection

Data on total number of live births at CHBAH during the study period were obtained from the CHBAH, Department of Obstetrics and Gynaecology records. We did not include number of live births from other facilities that referred neonates to CHBAH as this information was not available. All positive microbiological cultures identified from blood and/or CSF during the study period, including antibiotic susceptibilities, were retrieved from the National Health Laboratory Services (NHLS) database and captured for analysis. This retrospective data from the 10-year study period were retrieved and captured for analysis between October 2020 and June 2021. Blood and CSF isolates were combined during data capturing as either blood or CSF and therefore could not be separated for analysis. Clinical patient identification and information, such as demographics, clinical presentation and timing of onset, risk factors and outcomes as well as ancillary laboratory and antifungal susceptibility data are only collected on paper notes and were not available for this retrospective analysis (there is no electronic medical records available as in most LMIC settings). Duplicate or new isolates from the same patient could not be differentiated.

### Study definitions

Infants were defined as all patients admitted in the neonatal unit at birth or during the neonatal period and remained an in-patient at the time of blood or CSF culture sampling. Patient-days were reported as the sum of the daily number of occupied hospital beds. CoNS, *Corynebacteria* and *Bacillus spp*. isolates were not considered as clinically significant, and were excluded from the analysis. At our site a very small proportion of CoNS isolates (approximately 5%), are considered pathogenic, and the majority of CoNS isolates are not routinely treated with antibiotics [35]. If an organism, excluding CoNS, *Corynebacteria* and *Bacillus spp*., was isolated from blood and/or CSF, it was considered a significant infection. *Streptococcus viridans* were included in the analysis as pathogens as they have often been responsible for significant, invasive infections in infants admitted in our neonatal unit, requiring antimicrobial treatment. Infection rate was calculated by dividing the total number of isolates from blood and/or CSF by the total number of patient-days or live-births and multiplying by 1000. MDRO were defined as isolates with reduced susceptibility to at least one antibiotic in three or more of the five major antimicrobial classes [36]. Extended spectrum beta-lactamase (ESBL) organisms were defined as those that produce ESBL enzymes that break down and confer resistance to commonly used beta-lactam antibiotics. Carbapenem resistance was defined as intermediate or complete resistance to one or more of the tested carbapenem antibiotics, namely meropenem, imipenem and ertapenem [36].

### Statistical analysis

Data retrieved from the NHLS was captured into a Microsoft-Excel (Microsoft Corporation One Microsoft Way Redmond, WA 98052–6399. USA) spreadsheet. Statistical analysis was performed using STATA, version 17 (StataCorp LLC 4905 Lakeway Drive College Station, Texas 77845–4512. USA). Overall and pathogen-specific blood and/or CSF infection rates were calculated and presented as number of blood and/or CSF isolates/1000 patient-days, the number of blood and/or CSF isolates/1000 live-births and as a proportion of the number of admissions. Proportions of the different types of pathogens, and multidrug resistance within these isolates, were calculated out of the total isolates cultured. Trends in numeric variables and rates (e.g., infection rates) over the 10-year period were assessed using linear regression and logistic regression, respectively, fitting year as a continuous linear variable. Results are presented as estimated average yearly change with associated 95% confidence intervals.

### Ethical considerations

The approval to conduct this study was obtained from the Hospital Chief Executive Officer and the University of the Witwatersrand Human Research Ethics Committee. A waiver of informed consent was granted due to the retrospective nature of the study (Ethics reference number: M200236)

## Results

### Bloodstream and CSF infection rates

There were 206,644 hospital live births between 1^st^ January 2010 and 31^st^ December 2019, with 39,391 admissions to the neonatal unit and a total of 582,065 patient-days. Excluding CoNS (n = 2142; 20%), a total of 8,319 blood and/or CSF isolates were identified, accounting for 21.1% of admissions and giving an overall infection rate of 40.3/1000 live-births and 14.3/1000 patient-days (Table 1).

**Table 1:** Annual number of isolates, live births, admissions and patient-days, and annual infection rates from 2010 to 2019.

Year	Number of isolates	Number of live births	Number of admissions	Number of patient-days	Rates of infections (/1000 live births)	Proportion of admissions with isolates from blood and/or CSF sites (%)	Rates of infections (/1000 patient days)
2010	616	22657	3417	51246	27.2	18.0	12
2011	616	22803	4130	53473	27.0	14.9	11.5
2012	636	21588	4308	56497	29.5	14.8	11.3
2013	626	22288	4318	58159	28.1	14.5	10.7
2014	889	20524	4160	57303	43.3	21.4	15.5
2015	974	19804	4326	58223	49.2	22.5	16.7
2016	1149	19219	3071	59929	59.8	37.4	19.2
2017	1067	20575	4335	63429	51.9	24.6	16.8
2018	804	18758	3891	63778	42.9	20.7	12.6
2019	942	18428	3435	60028	51.1	27.4	15.7
**Total**	**8319**	**206644**	**39391**	**582065**	**40.3**	**21.1**	**14.3**

**Fig 1:**
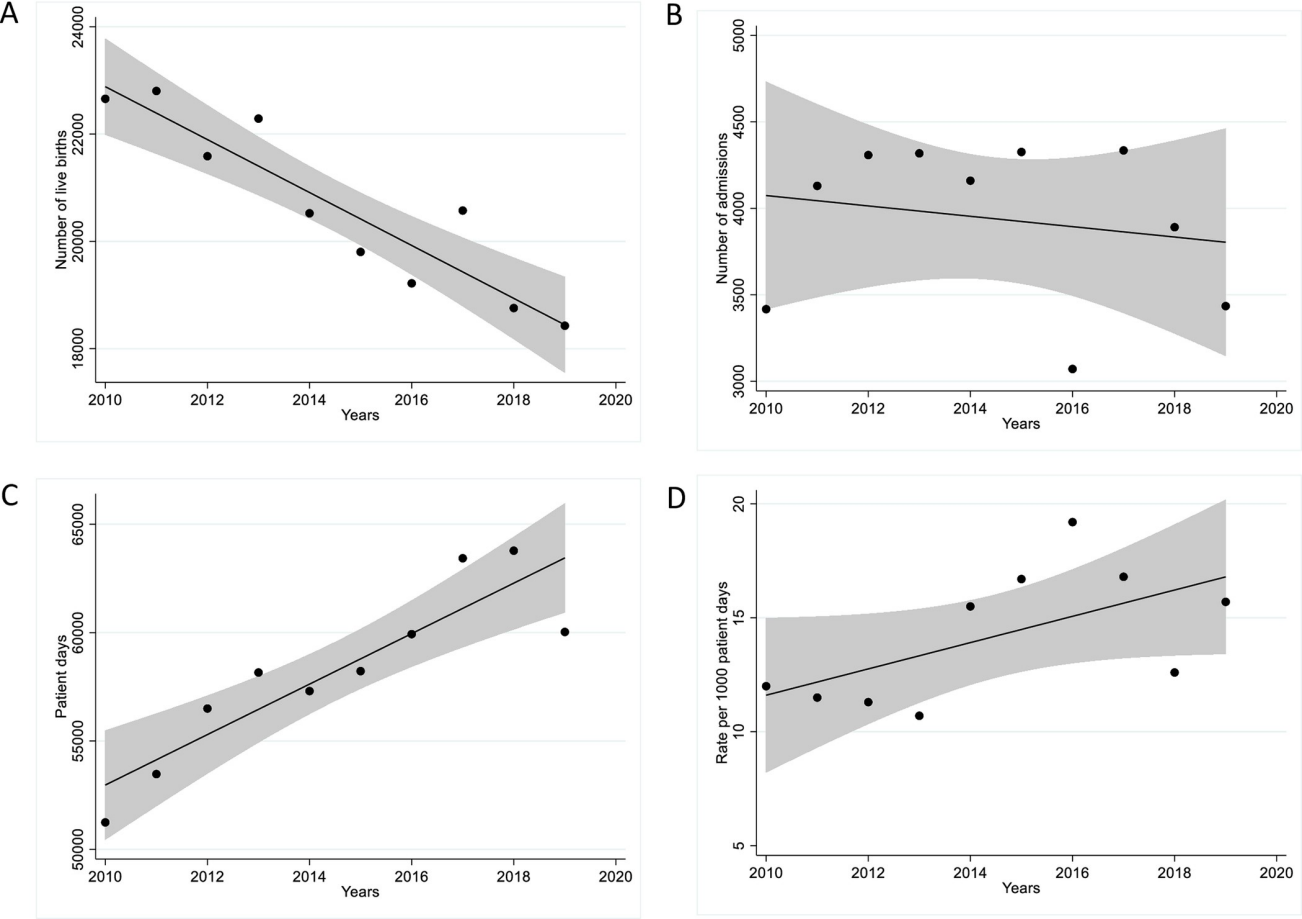
Trends in A. live births, B. annual admissions, C. patient-days and D. blood and/or CSF isolates per 1000 patient days, from 2010 to 2019.

Though the number of live-births decreased slightly by an average of 493 yearly ([95% CI, 326–660]; p = <0.001), the number of admissions remained unchanged with 3,417 and 3,435 admissions in 2010 and 2019 respectively (estimated average yearly change -30 [95%CI, -153-93]; p = 0.589). Patient-days increased, by a yearly average of 1,164 ([95%CI, 694–1,634]; p<0.001) with patient-days in 2019 being 17% higher than in 2010 (Fig 1A–1C). From 2010 to 2019, the incidence rates of infection increased from 12.0 to 15.7/1000 patient-days (estimated average yearly change, 0.6[95%CI, 0.5–0.7]; p = <0.001). Infection rates per number of live-births increased by almost 90% from 27.2 to 51.1/1000 live-births (estimated average yearly change, 3.3[95%CI, 3.0–3.6]; p = <0.001), with the lowest infection rate of 10.7/1000 patient-days seen in 2013 and the highest infection rate of 19.2/1000 patient-days seen in 2016. The proportion of admissions also increased by 50% from 18.0% to 27.4% (estimated average yearly change, 1.5% [95%CI, 1.3%– 1.6%]; p = <0.001) (Fig 1D).

### Proportion and rates of infection due to different pathogen types

**Fig 2:**
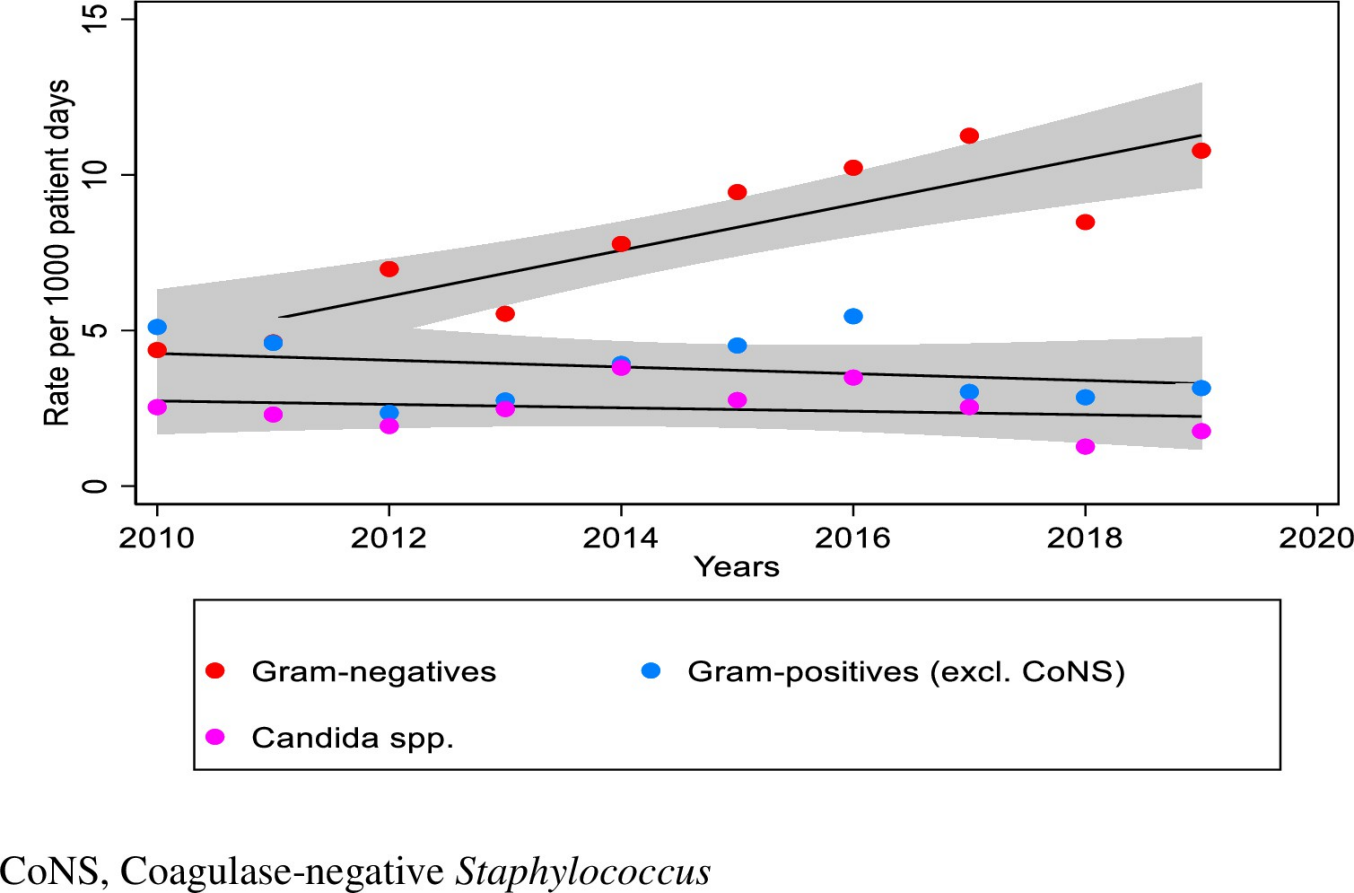
Rates of blood and/or CSF infections for different pathogens over the 10-year period.

Bacteria accounted for 82.7% of all isolates. Overall, Gram-negative isolates predominated (n = 4,698; 57%), followed by Gram-positive (n = 2,179; 26%) and fungal isolates (n = 1,442; 17%). Gram-positive organisms were marginally the commonest organisms isolated in 2010 and thereafter Gram-negative isolates increasingly predominated from 2011 onwards (Table 2). Rates of Gram-negative infections increased from 4.3/1000 patient-days in 2010 to 10.8/1000 patient-days in 2019 (estimated average yearly change 0.7[95%CI, 0.6–0.8]; p = <0.001). Rates of Gram-positive infections steadily decreased (estimated average yearly change -0.1[95%CI, -0.2 –-0.05]; p = <0.001). Gram-negatives as a proportion of all bacterial isolates increased from 46% in 2010 to 77% in 2019 (estimated average yearly change 2.7% [95%CI, 2.3%– 3.1%]; p = <0.001), with the Gram-negative to Gram-positive ratio increasing substantially from 0.9 in 2010 to 3.4 in 2019. Rates of fungal infections decreased slightly from 2.5/1000 patient-days in 2010 to 1.8/1000 patient-days in 2019 (estimated average yearly change -0.06[95%CI, -0.1 –-0.02]; p = 0.007) (Fig 2).

**Table 2:** Annual and total number and proportion of Gram-positive, Gram-negative and fungal isolates from 2010 to 2019.

	Gram Positives n (%)	Gram Negatives n (%)	Fungi n (%)	TOTAL
**2010**	262 (43)	224 (36)	130 (21)	616
**2011**	246 (40)	247 (40)	123 (20)	616
**2012**	133 (21)	394 (62)	109 (17)	636
**2013**	160 (26)	322 (51)	144 (23)	626
**2014**	225 (25)	446 (50)	218 (25)	889
**2015**	263 (27)	550 (56)	161 (17)	974
**2016**	327 (29)	613 (53)	209 (18)	1149
**2017**	192 (18)	714 (67)	161 (15)	1067
**2018**	182 (23)	541 (67)	81 (10)	804
**2019**	189 (20)	647 (69)	106 (11)	942
Total	2179 (26)	4698 (56)	1442 (17)	8319

**Fig 3:**
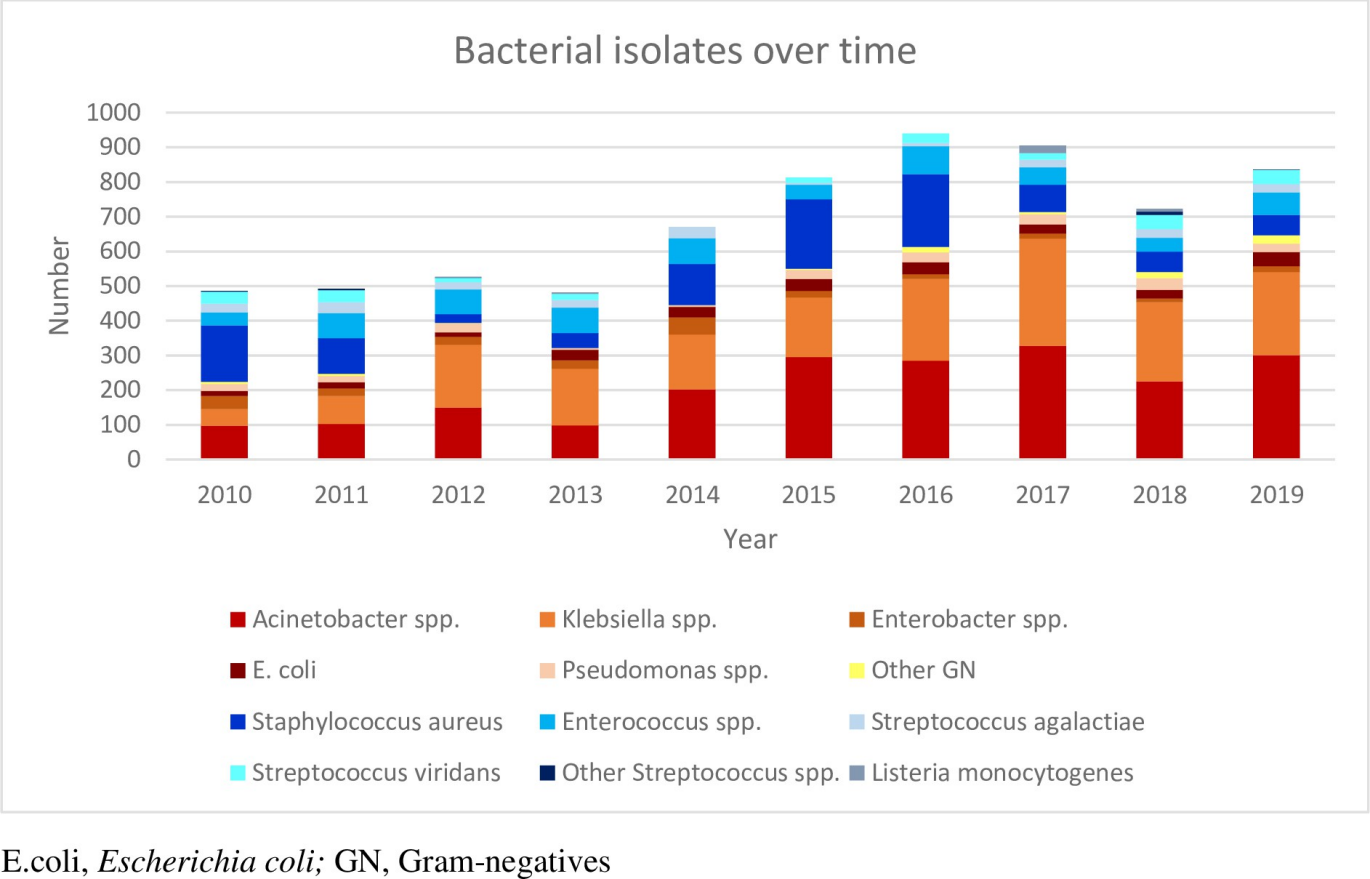
Bacterial isolates from blood and/or CSF, per year, from 2010 to 2019.

The most commonly isolated Gram-negative organisms were *Acinetobacter baumannii* (2,072; 44%), *Klebsiella pneumoniae* (1,794; 38%), *Escherichia coli* (266; 6%), *Enterobacter cloacae*. (219; 5%) and *Pseudomonas aeruginosa* (215; 4%) (Table 3). Among the Gram-positive organisms *Staphylococcus aureus* (1,057; 48%), *Enterococcus faecalis (360; 17%)*, *Streptococcus viridans* (241; 11%), *Enterococcus faecium* (220; 10%) and *Streptococcus agalactiae* (220; 10%) were the predominant species. Fig 3 shows the number of bacterial isolates cultured during the study period. Among the fungal isolates, *Candida parapsilosis* (758; 52%) and *Candida albicans* (501; 35%) were most commonly isolated.

**Table 3:** Profile of the different bacterial and fungal isolates cultured from blood and/or CSF per year, from 2010–2019.

	2010 (n = 616)	2011 (n = 616)	2012 (n = 636)	2013 (n = 626)	2014 (n = 889)	2015 (n = 974)	2016 (n = 1149)	2017 (n = 1067)	2018 (n = 804)	2019 (n = 942)	Total (N = 8319)
	n (%)	n (%)	n (%)	n (%)	n (%)	n (%)	n (%)	n (%)	n (%)	n (%)	N (%)
**Gram-Negatives**	**224 (36)**	**247(40)**	**394(62)**	**322(51)**	**446(50)**	**550(56)**	**613(53)**	**714(67)**	**541(67)**	**647(69)**	**4698 (56)**
*Acinetobacter baumannii*	*93 (42)*	*103 (42)*	*150 (38)*	*99 (31)*	*202 (45)*	*287 (52)*	*285 (46)*	*327 (46)*	*225 (42)*	*301 (46)*	2072 (44)
*Acinetobacter lwoffii*	*5 (2)*	*0 (0)*	*0 (0)*	*0 (0)*	*0 (0)*	*9 (2)*	*0 (0)*	*1 (0)*	*0 (0*.*1)*	*0 (0)*	15 (0.3)
*Klebsiella pneumoniae*	*45 (20)*	*77 (31)*	*176 (45)*	*158 (49)*	*156 (35)*	*171 (31)*	*235 (38)*	*307 (43)*	*229 (42)*	*240 (37)*	1794 (38)
*Klebsiella oxytoca*	*3 (1)*	*3 (1)*	*5 (1)*	*5 (2)*	*3 (1)*	*0 (0)*	*1 (0*.*2)*	*2 (0*.*3)*	*0 (0)*	*0 (0)*	22 (1)
*Pseudomonas aeruginosa*	*20(9)*	*18(7)*	*26(7)*	*5(1)*	*5(1)*	*26(5)*	*28(5)*	*29(4)*	*34(6)*	*24(4)*	215 (4)
*Escherichia coli*	*14(6)*	*17(7)*	*13(3)*	*31(10)*	*30(7)*	*35(6)*	*34(6)*	*26(4)*	*25(5)*	*41(6)*	266 (6)
*Enterobacter cloacae*	*30 (13)*	*20 (8)*	*23 (6)*	*22 (7)*	*49 (11)*	*19 (3)*	*14 (2)*	*15 (2)*	*10 (2)*	*17 (3)*	219 (5)
*Enterobacter spp*. *unspecified*	*8 (4)*	*3 (1)*	*0 (0)*	*2 (0*.*9)*	*0 (0)*	*0 (0)*	*0 (2)*	*0 (0)*	*0 (0)*	*0 (0)*	13 (0.3)
*Others* ^*#*^	*6(3)*	*6(2)*	*1(0)*	*0(0)*	*1(0)*	*3(1)*	*16(3)*	*7(1)*	*18(3)*	*24(4)*	82 (2)
											
**Gram-Positives**	**262 (43)**	**246(40)**	**133(21)**	**160(26)**	**225(25)**	**263(27)**	**327(29)**	**192(18)**	**182(23)**	**189(20)**	**2179 (26)**
*Staphylococcus aureus*	*163(62)*	*103(42)*	*25(19)*	*43(27)*	*118(52)*	*200(76)*	*210(64)*	*78(41)*	*59(32)*	*58(31)*	1057 (48)
*Enterococcus faecalis*	*13 (5)*	*24 (10)*	*31 (23)*	*57 (36)*	*44 (20)*	*5 (2)*	*62 (19)*	*33 (17)*	*39 (21)*	*52 (28)*	360 (17)
*Enterococcus faecium*	*19 (7)*	*41 (17)*	*36 (27)*	*13 (8)*	*26 12)*	*37 (14)*	*18 (5)*	*17 (9)*	*0 (0)*	*13 (7)*	220 (10)
*Enterococcus spp*. *unspecified*	*6 (2)*	*7 (2)*	*5 (4)*	*3 (2)*	*4 (1)*	*0 (0)*	*0 (0)*	*1 (1)*	*0 (0)*	*0 (0)*	26 (1)
*Streptococcus agalactiae*	*25(10)*	*32(13)*	*21(16)*	*22(14)*	*33(15)*	*6(2)*	*10(3)*	*22(11)*	*25(14)*	*24(13)*	220 (10)
*Streptococcus viridans*	*34(13)*	*34(14)*	*12(9)*	*18(11)*	*0(0)*	*15(6)*	*27(8)*	*19(10)*	*41(23)*	*41(22)*	241 (11)
*Streptococcus spp*. *unspecified*	*2(1)*	*5(2)*	*1(1)*	*0(0)*	*0(0)*	*0(0)*	*0(0)*	*0(0)*	*10(5)*	*1(1)*	19 (1)
*Listeria monocytogenes*	*0(0)*	*0(0)*	*2(1)*	*4(2)*	*0(0)*	*0(0)*	*0(0)*	*22(11)*	*8(5)*	*0(0)*	36 (2)
* *	* *	* *	* *	* *	* *	* *	* *	* *	* *	* *	
**Fungi**	**130 (21)**	**123(20)**	**109(17)**	**144(23)**	**218(25)**	**161(17)**	**209(18)**	**161(15)**	**81(10)**	**106(11)**	**1442 (17)**
*Candida parapsilosis*	*66(51)*	*61(49)*	*59(54)*	*74(51)*	*78(36)*	*103(64)*	*120(57)*	*102(63)*	*47(58)*	*48(45)*	758 (52)
*Candida albicans*	*45(35)*	*56(46)*	*31(29)*	*48(33)*	*112(51)*	*44(27)*	*76(36)*	*45(28)*	*27(33)*	*17(16)*	501 (35)
*Candida auris*	*0(0)*	*0(0)*	*0(0)*	*0(0)*	*0(0)*	*0(0)*	*0(0)*	*1(1)*	*0(0)*	*21(20)*	22 (2)
*Candida glabrata*	*15(11)*	*6(5)*	*11(10)*	*21(15)*	*23(11)*	*11(7)*	*8(4)*	*10(6)*	*6(7)*	*15(14)*	126 (9)
*Others* *	*4(3)*	*0(0)*	*8(7)*	*1(1)*	*5(2)*	*3(2)*	*5(2)*	*3(2)*	*1(1)*	*5(5)*	35 (2)

spp., species.

^*#*^ *Serratia marcescens*, *Proteus mirabilis*, *Stenotrophomonas maltophilia*, *Proteus mirabilis*, *Morganella morganii*, *Haemophilus influenzae*, *Citrobacter spp*., *Burkolderia spp*., *Alcaligenes spp*.

^*^Candida glabrata, *Candida krusei*, *Candida lusitaniae*, *Candida tropicalis*.

### Susceptibilities of common Gram-negative isolates

**Fig 4:**
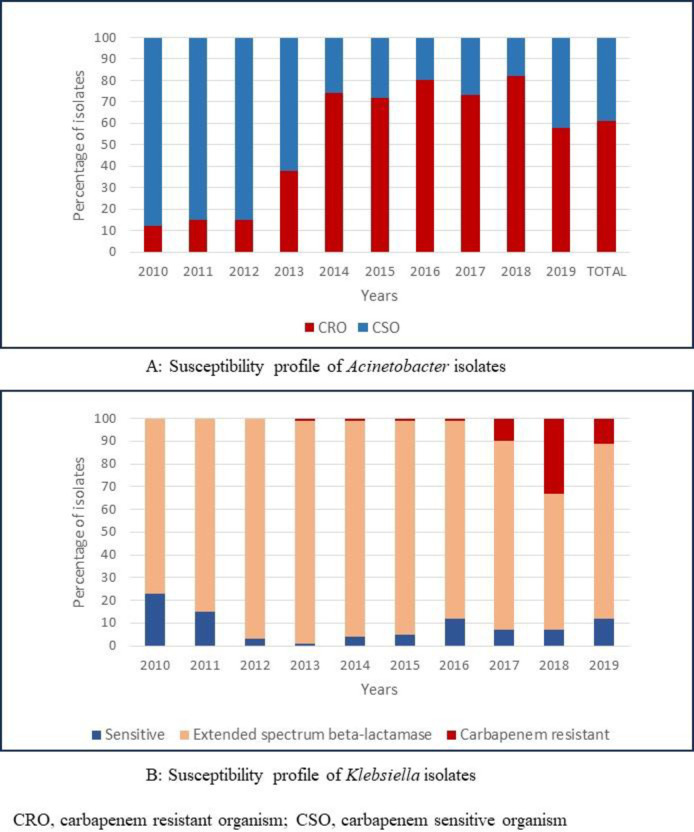
Proportion of *Acinetobacter* and *Klebsiella* isolates cultured from blood and/or CSF that were carbapenem resistant, from 2010 to 2019.

Of all 2,087 *Acinetobacter* isolates, 1,277 (61%) were classified as carbapenem resistant (Fig 4). The proportion of carbapenem resistant isolates increased from 12% in 2010, to 58% in 2019 (estimated average yearly change 6.8% [95%CI, 5.8%-7.7%], p< 0.001).

The great majority (n = 1,539; 85%) of the 1,816 *Klebsiella* isolates were ESBL organisms and 8% (n = 138) were carbapenem resistant (CR-*Klebsiella*). There was an average yearly decline of the proportion of ESBL isolates of 3.1% ([95%CI, 3.7% - 2.5%]; p<0.001). The first CR-*Klebsiella* was observed in 2013, accounting for 1% of *Klebsiella spp*. in 2013, and this increased to 11% in 2019 (estimated average yearly change 2.1% [95%CI, 1.8%-2.5%]; p<0.001). Overall, carbapenem resistance was seen in 31% (1469/4698) of all Gram-negative isolates and increased over time (estimated average yearly change 4.8% [95%CI, 4.2%-5.3%]; p<0.001)

### Susceptibilities of common Gram-positive isolates

**Fig 5:**
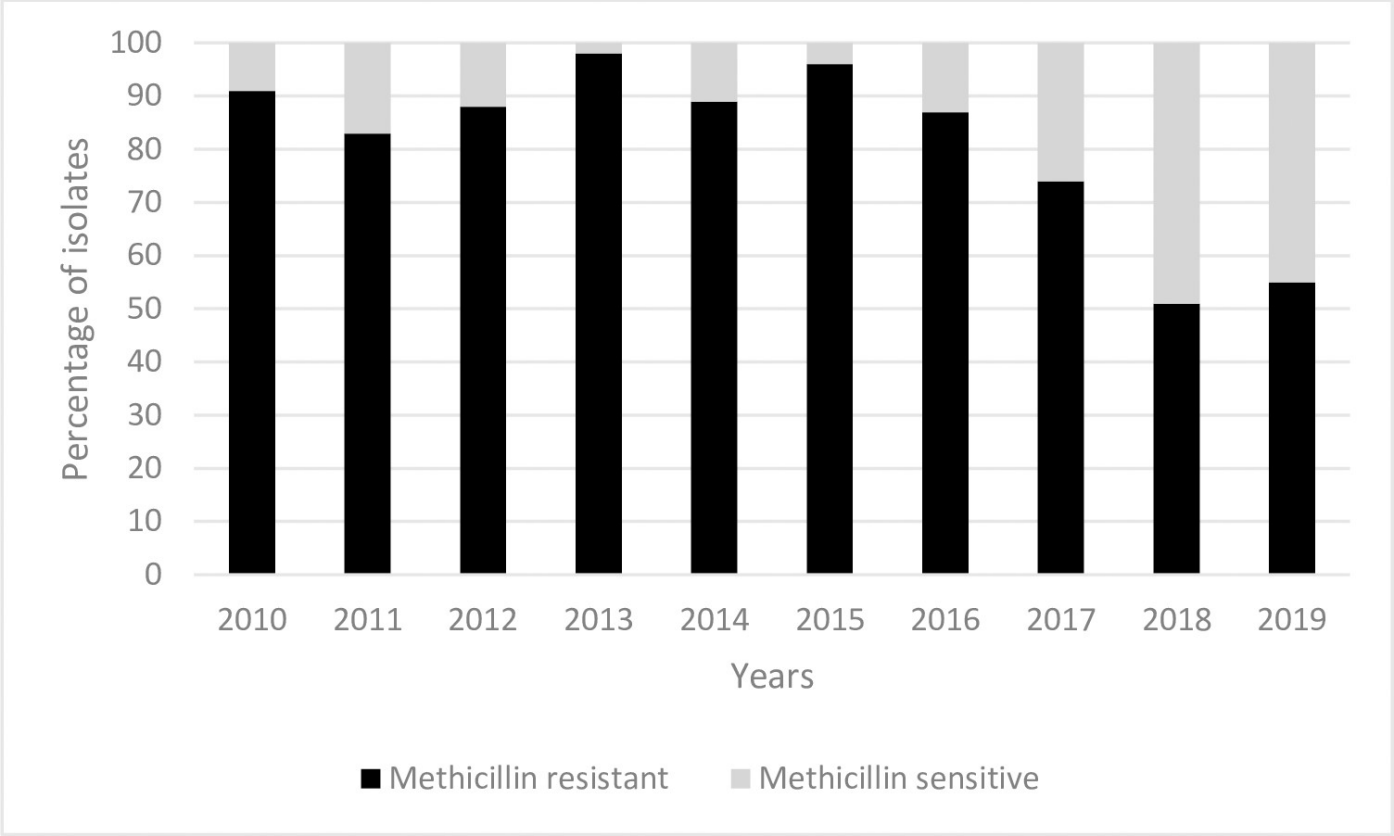
Proportion of *Staphylococcus aureus* isolates with methicillin resistance cultured from blood and/or CSF, from 2010 to 2019.

*Staphylococcus aureus* was the most common Gram-positive organism isolated. Of the 1,057 *Staphylococcus aureus* isolates, 898 (85%) were methicillin resistant. Rates of methicillin resistant *Staphylococcus aureus* (MRSA) decreased from 91% in 2010 to 55% in 2019 (estimated average yearly change -2.8% [95%CI, -3.5% –2%]; p<0.001) (Fig 5).

## Discussion

In this 10-year retrospective review of positive blood and CSF culture isolates, we observed a high incidence of culture positive bloodstream and/or CSF infections in infants admitted in the neonatal unit at CHBAH. Overall, Gram-negative pathogens predominated, with a shift from predominantly Gram-positive organisms in 2010 to predominantly Gram-negative organisms in 2019. *Acinetobacter baumannii* and *Klebsiella pneumoniae* were the most commonly isolated Gram-negative organisms. Though rates of ESBL *Klebsiella* remained consistently high at >80%, increasing rates of CR-*Klebsiella* were noted, from 0% in 2010 to 11% in 2019. Rates of carbapenem resistant *Acinetobacter* more than quadrupled from 12% in 2010 to 58% in 2019. *Staphylococcus aureus* was the most commonly isolated Gram-positive organism, but the proportion of MRSA decreased steadily over time. Among the fungi, *Candida parapsilosis* predominated, followed by *Candida albicans*, with an increase in *Candida auris* noted in 2019.

A reduction in the number of live births was noted, but the number of annual admissions remained unchanged. This is likely explained by the opening of a new district hospital in Soweto, with obstetric facilities but with limited neonatal admission facilities. Despite the unchanged number of annual admissions, number of patient-days increased, likely due to improved resources and advancements in neonatal care resulting in improved survival of very low birth weight infants and longer hospital-stay. In addition, the increasing trends seen in infection rates over the years would also contribute to increased length of hospital stay and consequently increasing patients-days. Clinical data on birth weights and length of hospital stay over the study period was not available for analysis.

The incidence of culture confirmed blood and/or CSF infections in this study is very high at 14.3/1000 patient-days compared to approximately 3/1000 patient-days reported in HIC, despite most of these studies including CoNS, which we considered as contaminant [37–39]. This incidence is also higher than that reported from other LMIC [40–42], including those from other facilities in South Africa [32,43,44]. A recent retrospective 10-year review of neonatal HAI in a tertiary hospital in Western Cape, South Africa reported an overall BSI rate of 3.6/1000 patient-days, and a decrease in rates over time [32]. This is in contrast to this study which showed an increase in infection rates from 12/1000 patient-days in 2010 to 15.7/1000 patient-days in 2019. However, our study included all positive blood and CSF cultures from hospital admission, unlike the Western Cape study which only included BSI after 3 days of admission.

We found an increasing predominance of Gram-negative organisms, comprising 69% of isolates in 2019. The Delhi Neonatal Infection Study (DeNIS), also reported the majority (75%) of isolates being Gram-negatives [4]. The Western Cape study reported on a Gram-negative predominance of 63.8% [32]. Another South African study reported on a predominance of Gram-positive organisms, at 68.7%, however it included CoNS, but with it excluded Gram-negative organisms predominated at 57%, similar to findings in this study [45]. Gram-negative organisms have been shown to predominate in LMIC compared to HIC, where Gram-positive organisms tend to predominate [4,44,46]. Both *Acinetobacter baumannii* and *Klebsiella pneumoniae* have emerged as leading pathogens in many neonatal units, responsible for many hospital outbreaks [4,25,45,47–49].

High level of AMR was seen among Gram-negative isolates. Overall, carbapenem resistance was 31%, and increased from 5.4% in 2010 to 36.5% in 2019. These rates are similar from the two previously reported South African studies [32,45]. Similar rates of CR-*Klebsiella* were reported by the DeNIS group, at 35% [4]. Numerous studies have reported on the emergence of carbapenem resistance in neonatal units [41,50]. A recent web-based survey, involving 39 neonatal units from 12 countries, looked at period prevalence of blood culture isolates and their resistance patterns. Carbapenem resistance rates ranged from 0% - 80%, with rates in South Africa reported at 39% [51]. The BARNARDS observational study of neonatal sepsis and AMR in LMIC reported that approximately 15% of Gram-negative isolates were carbapenem resistant [16,52]. The Global Neonatal sepsis Observational Study (NeoOBS) conducted over 11 countries, mostly LMIC, also reported high rates of AMR with 34% of empiric treatment regimens aiming to provide ESBL/Pseudomonal coverage [53].

*Staphylococcus aureus* was the most commonly identified Gram-positive pathogen, in keeping with other studies, with the exclusion of CoNS [4,32,54]. Eighty-five percent of *Staphylococcus aureus* isolates were methicillin resistant. The two South African studies also reported high rates of MRSA. The DeNIS study group, however, reported much lower rates of MRSA of only 35%. There has been a decline in MRSA over time, which is encouraging. A local policy of daily 0.2% chlorhexidine baths for hospitalized infants was introduced in 2014, which may explain this finding [55,56]. In adults, daily bathing with chlorhexidine impregnated washcloths was associated with a significant reduction in MDRO and HAI [57]. In addition, 2% chlorhexidine baths has been shown to reduce the incidence of bacteraemia significantly in critically ill children [58]. In the neonatal population, whole body bathing with 1% or 2% chlorhexidine baths has been shown to reduce MRSA infection and colonization, as well as reduced central line associated bloodstream infections [55,56,59].

The main limitation in this study is the retrospective study design, from a single center, only reporting on positive blood and/or CSF isolates, without accompanying patient, clinical and ancillary laboratory data. Data collected over the years from the laboratory onto the Excel database used for this analysis only included names of isolates, site of culture, month and year of sampling, and antimicrobial resistance. Since this is a retrospective analysis that spans 10 years, it was not feasible to extract additional clinical information from the laboratory database for every isolate. Therefore, case definitions of neonatal sepsis and duplicate pathogens, as well as duplicate blood and CSF isolates from the same patient, were not accounted for and consequently rates presented here could be overestimated. Blood and CSF isolates were combined and therefore could not be analysed separately. CoNS was excluded from the analysis as they are not usually considered clinically significant in our unit, supported by negative repeat cultures within 48 hours and negative biomarkers, and are not routinely treated with antibiotics. A small proportion of pathogenic CoNS isolates could have been excluded inadvertently, in the absence of clinical patient information, ancillary markers and duplicate culture results. However, since the local case definition of neonatal sepsis or protocols for work-up for sepsis remained the same over time limitations were consistent throughout the study period. Data on infection outbreaks during the study period was not available, therefore it is also possible that the increased rates in pathogens seen in the yearly data may be a reflection of periodic outbreaks as opposed to a baseline increase in infection rates. However, the consistency and magnitude of the patterns observed indicate a strong temporal trend and are the reason for reporting this study. The main strength of this study is that it reports rates and trends from a single large, tertiary LMIC neonatal unit and the microbiological analysis was conducted by a single, high-quality laboratory using CLSI criteria. We believe the variation in trends are not related to performance of laboratory but are true trends in rates of sepsis, continued pathogen replacement and AMR.

## Conclusions

Numerous infection prevention and control (IPC) measures were implemented during the study period, including hand hygiene training, training on standard and transmission-based precautions, improved medication safety and antimicrobial stewardship, environmental hygiene, safe use of medical equipmentt and external reviews by visiting experts. However, like many other LMIC neonatal units, high birth rates, overcrowding, inadequate isolation facilities and shortages in nursing and cleaning staff numbers have remained major challenges, hindering the impact of IPC measures. Ongoing infection surveillance and reporting of hospitalized infants from neonatal units in LMIC should be prioritized and implemented, to better inform and guide IPC measures, as well as inform future studies aimed at providing guidance for future recommendations on empiric treatment regimens for the treatment of MDRO. In the resource limited setting improving the evidence base of cost-effective interventions is critical to their implementation. Reversing the inexorable trend of higher rates of increasingly untreatable infections in LMIC neonatal units is now a global health priority.

## Supporting information

S1 File Dataset. (XLSX)
